# Suppressing meta-holographic artifacts by laser coherence tuning

**DOI:** 10.1038/s41377-021-00547-0

**Published:** 2021-05-19

**Authors:** Yaniv Eliezer, Geyang Qu, Wenhong Yang, Yujie Wang, Hasan Yılmaz, Shumin Xiao, Qinghai Song, Hui Cao

**Affiliations:** 1 Department of Applied Physics, Yale University, New Haven, CT 06520 USA; 2 Ministry of Industry and Information Technology Key Lab of Micro-Nano Optoelectronic Information System, Harbin Institute of Technology (Shenzhen), Shenzhen, 518055 P. R. China

**Keywords:** Displays, Semiconductor lasers, Metamaterials

## Abstract

A metasurface hologram combines fine spatial resolution and large viewing angles with a planar form factor and compact size. However, it suffers coherent artifacts originating from electromagnetic cross-talk between closely packed meta-atoms and fabrication defects of nanoscale features. Here, we introduce an efficient method to suppress all artifacts by fine-tuning the spatial coherence of illumination. Our method is implemented with a degenerate cavity laser, which allows a precise and continuous tuning of the spatial coherence over a wide range, with little variation in the emission spectrum and total power. We find the optimal degree of spatial coherence to suppress the coherent artifacts of a meta-hologram while maintaining the image sharpness. This work paves the way to compact and dynamical holographic displays free of coherent defects.

## Abstract (web summary)

A special type of laser enables precise and continuous tuning of spatial coherence, suppressing the coherent artifacts (i) of a meta-hologram while maintaining image sharpness, emission spectrum and power (ii).

## Graphical abstract

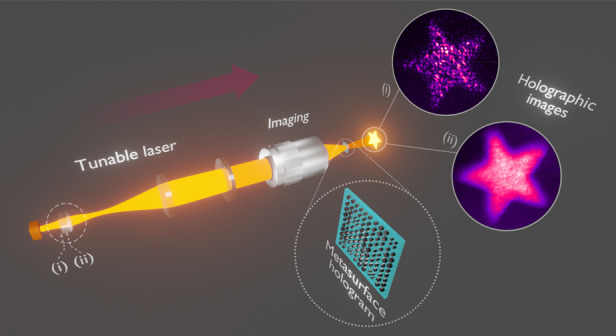

## Introduction

Artificial metasurfaces, comprised of a two-dimensional (2D) array of subwavelength scatterers, have shown unprecedented ability in controlling optical wavefront and converting conventional bulky optical elements into planar thin films^1–3^. One prominent example is the metasurface hologram (meta-hologram)^4–7^. An ultrathin metasurface is capable of reconstructing a three-dimensional (3D) holographic image with a high spatial resolution and large viewing angles, while suppressing high-order diffraction^8–12^. Very recently, multi-color, multiplexed, and dynamic meta-holograms have been proposed and demonstrated, illustrating a great potential in information processing, 3D display, high-density data storage, and optical image encoding^13–20^. Despite of these remarkable advances, the road to practical applications of meta-holograms is hindered by coherent artifacts. Such artifacts originate from near-field interactions of subwavelength scatterers (meta-atoms), fabrication defects and phase dislocations, causing image distortion and degradation^6,21^. While coherent artifacts and speckle noise are well-known issues for conventional holography, they are more significant in regard to meta-holography, as close packing of meta-atoms enhances the cross-talk and fabrication of nanoscale features is susceptible to error. Such artifacts cause severe distortions of holographic images, which are extremely difficult to correct. Recently, machine-learning-based optimization techniques have been applied to high-performance metasurface design^22–24^. They require a large amount of training data, which are difficult and expensive to acquire for large-scale meta-holograms. Moreover, the coherent artifacts caused by random fabrication imperfections cannot be removed by optimizing the meta-hologram design. An alternative way of suppressing coherent artifacts is adjusting the spatial and/or temporal coherence of illuminating light^25^. In general, it is more efficient to suppress speckle noise by reducing spatial coherence than temporal coherence^26^. While lowering the temporal coherence with broadband illumination provides spectral compounding, the different wavelengths generate different radial scalings of speckle patterns resulting in a deficient suppression of coherent artifacts^27^. Previously, lowering the spatial coherence of illumination has been widely used for the suppression of speckle noise in conventional holography^28^. It is done by either increasing the spatial coherence of a light-emitting-diode (LED) with spatial filtering^29–31^, or decreasing the spatial coherence of a laser with moving elements and time integration^32–35^. While the former technique suffers from severe power loss, the latter requires long exposure time. Since reducing the spatial coherence would blur the image and reduce the depth of field, a precise tuning of the spatial coherence is required.

To eliminate coherent artifacts of meta-holograms, we resort to a degenerate cavity laser (DCL) with tunable spatial coherence for illumination. The DCL provides a wide tuning range of spatial coherence with little power loss^36,37^. Its fast decoherence enables a short exposure time for high-speed imaging^38^. Furthermore, the emission spectrum of the DCL does not change during the spatial coherence tuning, avoiding the spectral dispersion of the meta-hologram. By fine-tuning the spatial coherence of the DCL, we find the optimal degree that suppresses all sorts of artifacts without a significant blurring of the holographic image. Our scheme works efficiently for different types of meta-holograms, providing a general method for artifact-free holographic display.

## Results

### Coherent artifacts created by meta-holograms

**Fig. 1:**
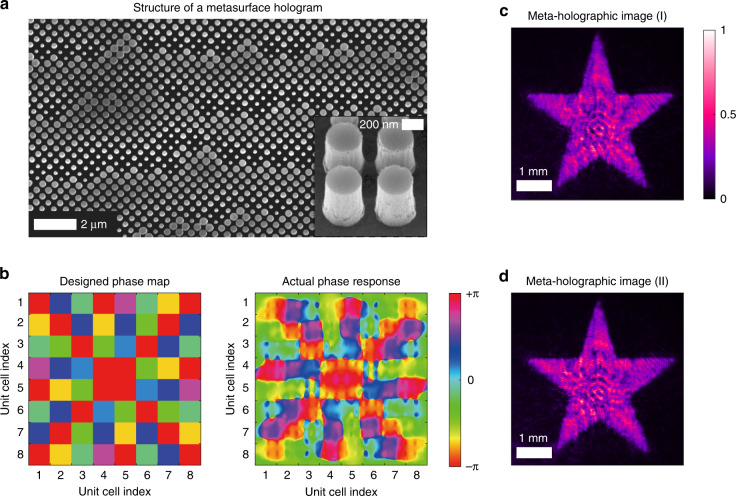
Coherent artifacts of metasurface hologram. **a** Scanning electron microscope (SEM) image of a part of a meta-hologram comprised of 128 × 128 unit cells, each having 2 × 2 silicon nanopillars of the same diameter *D*. The spatial phase modulation is achieved by varying *D* from 142 to 366 nm. Inset: magnified view of the silicon nanopillars revealing surface roughness which causes unwanted scattering and interference of light. **b** (left) Designed phase map *φ*_H_ of a small meta-hologram comprised of 8 × 8 unit cells, based on the calculated phase response of individual nanopillars with different diameters. **b** (right) Actual phase response *φ*_A_ from a numerical simulation of the entire metasurface, showing a significant deviation from the designed one due to near-field interactions among neighboring nanopillars. **c**, **d** Holographic images of a star object generated by two fabricated meta-holograms with the same design shown in (**a**). Their intensity fluctuations are nearly identical, indicating that the fluctuations result mainly from deterministic interactions among meta-atoms. Optical vortices are already eliminated from the computer-generated hologram. The illumination source is a monochromatic laser at wavelength *λ* = 1064 nm, which has a high spatial and temporal coherence

We design and fabricate a metasurface hologram as shown in Fig. 1a. It is made of Silicon nanopillars on top of a glass substrate (see “Methods” for the design and fabrication processes). The phase modulation of the meta-hologram is achieved via resonant scattering of individual nanopillars (meta-atoms). By tuning the pillar diameter *D*, the scattering resonance frequency is varied and the phase response *φ* at the illumination wavelength is changed. In the meta-hologram design, *φ*(*D*) is calculated for a single pillar with periodic boundary conditions. To create a holographic image in the far-field, the near-field phase pattern *φ*_H_ is computed with an iterative phase retrieval (IPR) algorithm (see “Methods” for details). Then, the inverse mapping function *D*(*φ*) determines the spatial variation of the nanopillar diameter *D* to obtain the designed phase distribution *φ*_H_.

Since the nanopillars are densely packed, the near-field interactions among neighboring nanopillars are significant. In the hologram design, the periodic boundary conditions used in the phase calculation *φ*(*D*) correspond to the assumption that all neighboring pillars have an identical diameter *D*. This is not true in the actual meta-hologram, where *D* varies spatially. The near-field interactions between nanopillars with different diameters differ from the ones with the same diameter. Such difference causes the actual phase response (*φ*_A_) to deviate from the designed one (*φ*_H_).

To reduce this deviation, we adopt a unit cell with 2 × 2 identical nanopillars, so that some of the neighboring pillars have the same diameter and their couplings better agree with the calculation with periodic boundary condition. However, the increase of the unit cell size reduces the maximal viewing angle to 99°. To keep a relatively large viewing angle, we refrain from further enlarging the unit cell. Albeit weaker than the case of single-pillar unit cell, the cross-talk effects are still strong for the 4-pillar unit cell, as confirmed by a numerical simulation of a small meta-hologram with 8 × 8 unit cells. The actual phase modulation *φ*_A_ in Fig. 1b is notably different from the designed pattern *φ*_H_. Such difference leads to a severe distortion of the holographic image, as observed experimentally in Fig. 1c. The seemingly-random intensity fluctuation is reproduced with another fabricated meta-hologram of identical design in Fig. 1d. Magnified images are available in the Supplementary Materials. Therefore, the artifacts are primarily due to deterministic cross-talk among the meta-atoms. The resulting contrast of intensity fluctuations in the meta-holographic image is ∼0.3, much higher than the typical speckle contrast of classical holographic images.

Such cross-talk is very difficult to amend, because the meta-hologram in Fig. 1a is comprised of 128 × 128 = 16,384 unit cells, i.e., in total 16,384 × 4 = 65,536 nanopillars (meta-atoms). To accurately account for the interactions among all nanopillars of varying size, the phase response of the entire metasurface has to be calculated, a task which is computationally demanding. Any iterative optimization of the hologram configuration requires simulating the entire metasurface repetitively, which is beyond standard computing capabilities. It is neither practical to apply machine learning to this case, as an extensive simulation of such large meta-holograms with varying parameters is needed to train an artificial neural network.

In addition to the cross-talk of meta-atoms due to their near-field interactions, there are two different sources for meta-hologram artifacts. Due to the subwavelength size of the Silicon pillars, structural defects are introduced unintentionally during the fabrication of meta-hologram, as seen in the inset of Fig. 1a. Such defects induce unwanted light scattering and interference, producing additional artifacts in the holographic image. Furthermore, optical vortices are generated in the holographic image due to the creation of phase dislocations in the design of digital hologram. Such defects can be eliminated by incorporating complementary algorithms in the IPR^39–43^ (see “Methods”). Alternatively, they are eliminated by full-field (amplitude and phase) modulation with a meta-hologram^44,45^.

### Degenerate cavity laser with tunable spatial coherence

**Fig. 2:**
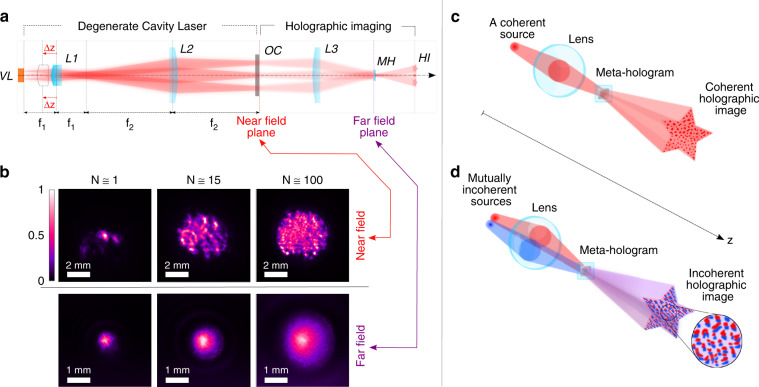
Tuning the spatial coherence with a degenerate cavity laser (DCL). **a** Schematic of the DCL comprising of a vertical external cavity surface emitting laser (VECSEL) module (VL), two lenses (L1, L2), and an output coupler (OC). The far-field DCL emission is projected by imaging optics (L3) onto the meta-hologram (MH), which creates a holographic image (HI) at its far-field. L1 is gradually translated along the cavity axis to break the cavity degeneracy condition, so that the number of transverse lasing modes *N* decreases and the spatial coherence of the total emission increases. **b** Near-field (top row) and far-field (bottom row) intensity patterns of the total emission with a varying number *N* of transverse lasing modes. With increasing *N*, there are more bright spots in the near-field (each corresponding to an independent lasing mode), and the far-field pattern becomes larger. **c** Schematic of a single lasing mode illuminating the meta-hologram, creating intensity fluctuations due to coherent artifacts. **d** Schematic of two mutually incoherent lasing modes illuminating the meta-hologram with different angles, creating laterally shifted and mutually incoherent holographic images. An incoherent (intensity) sum of the two images reduces the intensity fluctuations

To suppress coherent artifacts, we adjust the spatial coherence of illumination with a degenerate cavity laser (DCL). The DCL has a self-imaging configuration^46^, as shown in Fig. 2a. Since many transverse modes have a nearly degenerate quality factor, they can lase simultaneously and independently to reduce the spatial coherence of the emission. By tuning the cavity away from the degenerate condition (see “Methods“), the number of transverse lasing modes decreases, and the degree of spatial coherence increases.

Figure 2b shows the near-field (top row) and far-field (bottom row) intensity patterns of the laser emission. The near-field patterns at the DCL output coupler consist of bright spots, each corresponding to a transverse lasing mode. As the cavity approaches the degenerate condition, the number of spots (modes) increases. The diffracted beams from neighboring spots do not interfere, indicating that the modes are mutually incoherent. The number of independent lasing modes *N* is estimated from the intensity contrast of a speckle pattern generated by a static diffuser placed outside the laser cavity (see “Methods” and Supplementary Materials). As *N* decreases from ∼300 to ∼1, the emission power is merely reduced by 40% from 108 mW to 64 mW (see Supplementary Materials). Furthermore, the emission spectrum remains approximately the same with a full-width-at-half-maximum (FWHM) of about 3 nm, indicating that the temporal degree of coherence does not change (see Supplementary Materials). This effectively avoids the influence of chromatic aberration.

In contrast to the spotted near-field pattern, the laser emission exhibits a smooth profile at the far-field. It is composed of an incoherent superposition of Gaussian beams propagating in slightly different directions from individual spots at the near-field. The smooth intensity distribution ensures a homogeneous illumination of the meta-hologram which is placed at the far-field of the DCL.

The holographic image is created in the far-field of the meta-hologram. In the case of coherent illumination, the emission from a single transverse lasing mode illuminates the meta-hologram, as sketched in Fig. 2c. With partially coherent illumination in Fig. 2d, mutually incoherent lasing modes illuminate the meta-hologram with different angles of incidence and generate holographic images that are laterally shifted at the far-field. The number of distinct images is given by the effective number of independent spatial modes *N*_E_ in illumination, which is equal to the ratio of the area of the hologram to the coherence area of the illuminating light (see “Methods”). An intensity sum of *N*_E_ images will average out the intensity fluctuations due to coherent artifacts. However, the averaging also blurs the image and impairs the spatial resolution. Hence, the degree of spatial coherence must be optimized to suppress coherent artifacts without significantly degrading the image resolution.

### Suppression of holographic artifacts

**Fig. 3:**
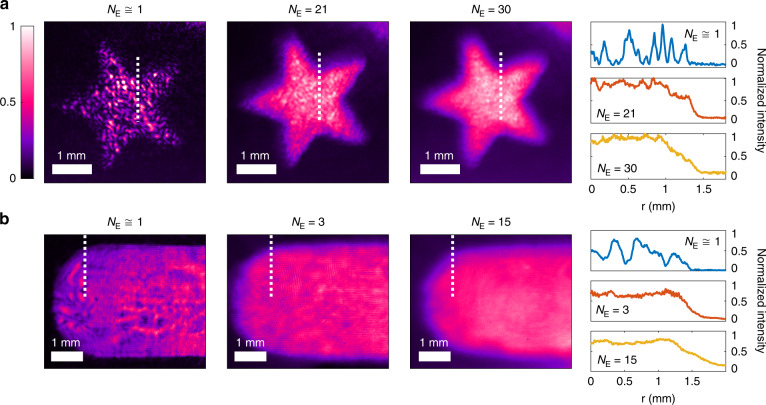
Meta-holographic images under varying spatial coherence DCL illumination. The meta-holograms are based on the resonant phase modulation in (**a**), and the geometric Pancharatnam-Berry (PB) phase in (**b)**. The coherent artifacts seen with high spatial coherence illumination (first column) gradually disappear, as the spatial coherence is lowered by increasing the effective number of spatial modes *N*_E_ that illuminate the meta-hologram (second column). Further decrease of the spatial coherence (increase of *N*_E_) notably blurs the image and reduces the edge sharpness (third column). The fourth column shows the 1D intensity profile through a cut of the holographic images marked by white dotted line in the first three columns

To demonstrate the capability of our method in suppressing all sorts of coherent artifacts, we design one set of meta-holograms with the standard Gerchberg-Saxton IPR algorithm^47^. The holographic images of this set contain many dark spots due to phase dislocations (optical vortices). The top row of Fig. 3 shows the holographic images, taken with varying degrees of spatial coherence of the DCL illumination. *N*_E_ is the effective number of spatial modes that illuminate the meta-hologram and generate laterally shifted holographic images. When the spatial coherence is high (*N*_E_ ≅ 1), the holographic image is full of coherent artifacts generated by near-field meta-atom interactions, fabrication defects, and phase dislocations. Lowering the spatial coherence by increasing *N*_E_ to 21 suppresses the intensity fluctuations, resulting in a nearly uniform holographic image. A further increase of *N*_E_ to 30, however, results in a notable reduction of the edge sharpness, as seen in the 1D intensity profile across an edge of the star image in Fig. 3.

We also test our method with meta-holograms free of phase dislocations. Despite the absence of optical vortices in the holographic images, intensity fluctuations are still significant, as seen in Fig. 1c, d. These artifacts can be strongly suppressed by optimizing the spatial coherence of the DCL illumination (see Supplementary Materials).

In addition to the resonant phase meta-holograms, our method is applicable to geometric Pancharatnam-Berry (PB) phase meta-holograms^1,48,49^. These holograms are also cleared of phase dislocations originating from the phase encoding process. A scanning electron microscope (SEM) image of the fabricated hologram is presented in the Supplementary Materials. The bottom row of Fig. 3 shows the holographic images recorded with the DCL illumination. Again, we observe intensity fluctuations under high spatial coherence illumination. In the absence of optical vortices, the artifacts result mainly from near-field interactions of meta-atoms. The intensity fluctuations are not as strong as those with optical vortices, thus a small decrease of the spatial coherence is sufficient to make the image smooth. The edges get blurred with a further reduction of the spatial coherence. The results of both types of meta-holograms confirm that the fine-tuning of the DCL spatial coherence is critical in achieving an optimal illumination condition where the holographic image is nearly free of coherent artifacts and remains relatively sharp.

### Optimal degree of spatial coherence

**Fig. 4:**
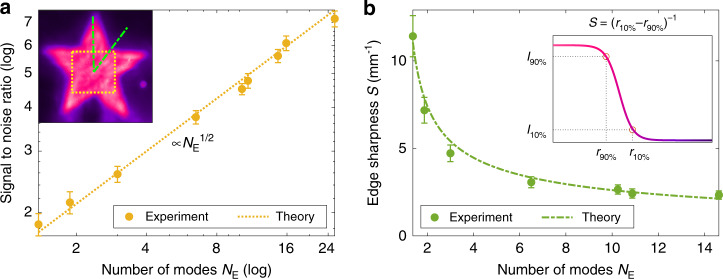
Quantitative assessment of a meta-holographic image quality. **a** Intensity signal to noise ratio (SNR) of a star image (inset) within the central square (marked by a yellow dotted line in the inset) versus the effective number of spatial modes *N*_E_ in illumination. Yellow circles are experimental data obtained by averaging the SNR over holographic images created by five meta-holograms. The error bars represent the standard deviation of the SNR. The data points follow the dotted yellow line with a slope of 1/2 in logarithmic scales, indicating that the SNR scales as $N_{\mathrm{E}}^{1/2}$. **b** Edge sharpness *S* as a function of the number of spatial modes *N*_E_. Green circles represent experimentally measured *S*, averaged over multiple edges of five holographic images. The error bars represent the standard deviation of *S*. The dotted-dashed green line denotes the theoretical prediction of our model. The inset illustrates how *S* is extracted from the 1D intensity profile across an edge (marked by a dotted-dashed green line in the inset of **a**)

To quantitatively assess the holographic image quality, we evaluate the signal-to-noise ratio (SNR) and edge sharpness. Experimentally, we collect the data of five meta-holograms free of phase dislocations. The meta-holograms generate the same holographic image of a star, as shown in the inset of Fig. 4a. The SNR is defined as $\left\langle I \right\rangle$*/σ*, where $\left\langle I \right\rangle$ is the average intensity within the central square marked in the inset of Fig. 4a, and *σ* is the standard deviation of the intensity fluctuation in this region. We average the SNR over five meta-holographic images, and plot its value versus the effective number of spatial modes *N*_E_. As seen in Fig. 4a, the SNR increases monotonically with *N*_E_. In logarithmic scales, the data points follow a straight line of slope 1/2, indicating that the SNR scales as $\sqrt {N_{\mathrm{E}}}$.

The edge sharpness is estimated from several 1D intensity profiles of the holographic image across different edges, as marked by green dotted-dashed lines in the inset of Fig. 4a. The sharpness is evaluated by estimating the slope of the edge, between two intensity points corresponding to 10% and 90% of the maximum intensity, *S* = (*r*_10%_ − *r*_90%_)^−1^ (see the inset of Fig. 4b). Figure 4b shows the edge sharpness *S* averaged over multiple edges of five holographic images. As the effective number of spatial modes *N*_E_ grows, *S* drops monotonically. Next we model the dependence of *S* on *N*_E_. For *N*_E_ = 1, the edge intensity profile is given by convolution of the ideal step function with the point spread function (PSF) of the holographic imaging setup. The width *w*_P_ of the PSF determines the spatial resolution, and is inversely proportional to the lateral dimension of the meta-hologram. For *N*_E_ > 1, there are *N*_E_ laterally shifted holographic images created by the meta-hologram, and an incoherent summation of all images broadens the edge intensity profile. The broadening depends on the width of the DCL near-field emission pattern (Fig. 2b), and is proportional to $\sqrt {N_{\mathrm{E}} - 1}$. As a result of convolution, the sharpness is given by $S\left( {N_{\mathrm{E}}} \right) = C_0\left[ {w_{\mathrm{P}}^2 + C_1 \cdot \left( {N_{\mathrm{E}} - 1} \right)} \right]^{ - 1/2}$, where *C*_0_ is a prefactor, *C*_1_ is a scaling constant relating the lateral shift of a holographic image to the tilt of incident angle of an illuminating beam (see Supplementary Materials for a complete derivation). Once the values of *C*_0_ and *C*_1_ are determined by fitting the experimental data with the expression of *S*(*N*_E_), the theoretical prediction of the edge sharpness (green curve in Fig. 4b) captures the measured dependence of *S* on *N*_E_.

**Fig. 5:**
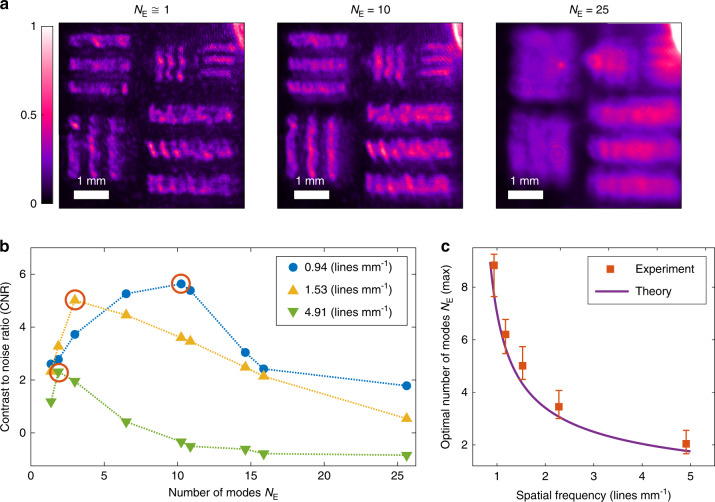
Optimal degree of spatial coherence. **a** Three holographic images of USAF test charts obtained with the same meta-hologram and illuminated by the DCL with varying degrees of spatial coherence. As the effective numbers of spatial modes *N*_E_ increases, the spatial coherence of the illumination decreases and the coherent artifacts are suppressed, however, the image resolution is impaired. **b** Contrast to noise ratio (CNR) versus the effective number of modes *N*_E_ for three spatial frequencies in the test charts. The CNR first increases with *N*_E_, reaches a maximum (circled in red) and then decreases. The maximum of the CNR shifts to a lower *N*_E_ for higher spatial frequencies (smaller feature size). **c** The optimal number of spatial modes $N_{\mathrm{E}}^{({\mathrm{max}})}$ at the maximal CNR decreases, as the spatial frequency (inverse of spatial resolution) increases. The purple curve is the theoretical prediction of our model

Finally, we identify the optimal degree of spatial coherence for illuminating a meta-hologram and find its dependence on the image resolution. To this end, we fabricate another set of meta-holograms that produce holographic images of a USAF resolution test chart. Figure 5a shows three holographic images with different degrees of spatial coherence illumination. The image quality is assessed by the contrast to noise ratio (CNR), which is defined as

1 $${\mathrm{CNR}} = \frac{{\left\langle {I_{\mathrm{S}}} \right\rangle - \left\langle {I_{\mathrm{B}}} \right\rangle }}{{\sigma _{\mathrm{S}}}} = \frac{{1 - \left\langle {I_{\mathrm{B}}} \right\rangle /\left\langle {I_{\mathrm{S}}} \right\rangle }}{{\sigma _{\mathrm{S}}/\left\langle {I_{\mathrm{S}}} \right\rangle }}$$

where $\left\langle {I_{\mathrm{S}}} \right\rangle$ and *σ*_S_ denote the average intensity and the standard deviation of the intensity fluctuation in the bright region, respectively. $\left\langle {I_{\rm{B}}} \right\rangle$ is the average intensity of the dark background. The numerator of the CNR, 1 − $\left\langle {I_{\mathrm{B}}} \right\rangle /\left\langle {I_{\mathrm{S}}} \right\rangle$, gives the intensity contrast between bright and dark regions, while the denominator $\sigma _{\mathrm{S}}/\left\langle {I_{\mathrm{S}}} \right\rangle$ characterizes the intensity fluctuation (noise) in the bright region. Overall, the CNR describes the resolvability of a feature of interest (bright) in a given background (dark).

Figure 5b shows the measured CNR varying non-monotonically with the effective number of spatial modes *N*_E_ for three different feature sizes. As *N*_E_ increases, the CNR first grows and then drops. It reaches a maximal value at an intermediate *N*_E_, indicating that there is an optimal degree of spatial coherence for illumination. When the feature size is large, the CNR reaches the maximum at a relatively large *N*_E_. Since the intensity contrast (in the numerator of CNR) remains high for a relatively wide range of *N*_E_, the CNR is determined mainly by the intensity fluctuation (in the denominator), which is smaller at larger *N*_E_. As the feature size decreases, the maximum CNR shifts to a lower *N*_E_. That is because, in resolving small features, the intensity contrast becomes more significant and is higher at smaller *N*_E_ due to less blurring. However, the maximal value of the CNR is less than that for a large feature size, because the intensity fluctuations are stronger. Therefore, the optimal degree of spatial coherence increases with the image resolution.

Figure 5c shows the optimal number of independent spatial modes $N_{\mathrm{E}}^{({\mathrm{max}})}$ (number of laterally shifted holographic images) required to reach the maximum CNR as a function of the spatial frequency (inverse of spatial resolution) in the USAF test chart. As the spatial frequency increases, the feature size decreases, and $N_{\mathrm{E}}^{({\mathrm{max}})}$ drops. Our theoretical modeling of the CNR (see Supplementary Materials) predicts $N_{\mathrm{E}}^{({\mathrm{max}})}$ (purple line) in good agreement with the experimental data.

## Discussion

**Fig. 6:**
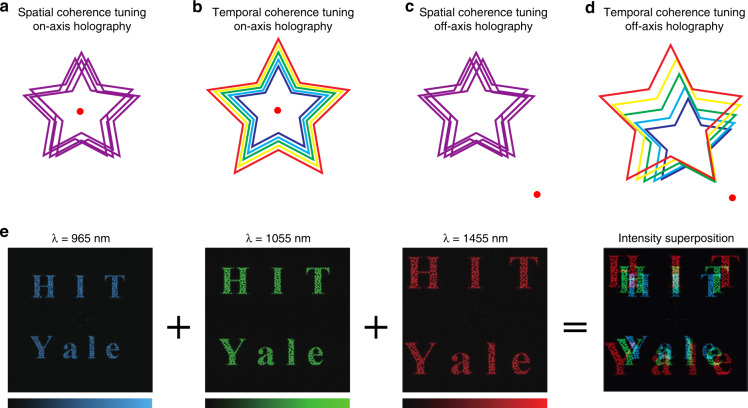
Comparison of spatial and temporal coherence tuning on holographic image quality. **a**–**d** Schematic of holographic images created by illuminating light with low spatial coherence and high temporal coherence (**a**, **c**), or with low temporal coherence but high spatial coherence (**b**, **d**) for on-axis holography (**a**, **b**) and off-axis holography (**c**, **d**). The optical axis is marked by a red dot. Lowering the temporal coherence results in stronger and non-uniform edge blurring than lowering the spatial coherence. **e** Numerically calculated meta-holographic images created by plane wave illumination at wavelengths *λ* = 965 nm, 1055 nm, 1455 nm. Pseudo-colors are used to label the wavelengths. The image size increases with wavelength, and an intensity sum of these images produces a radially extended image

In this work, we tune the spatial coherence of a degenerate cavity laser (DCL) to suppress strong coherent artifacts created by metasurface holograms. Compared to the conventional method of lowering the spatial coherence of a laser by a moving diffuser, our approach has several distinct advantages: (i) The precise, continuous tuning of the DCL spatial coherence allows to reach the maximal contrast to noise ratio (CNR) for any desired spatial resolution. (ii) The tuning is energy efficient, and does not introduce a significant power variation. (iii) The spectral width of the DCL emission (degree of temporal coherence) remains constant during the spatial coherence tuning, which is important for meta-holograms with strong dispersion. (iv) No pre- or post-processing procedures are needed in our method for coherent artifacts suppression. (v) Fast lasing dynamics leads to rapid decoherence of the DCL emission, thus enabling high-speed meta-holography. In comparison to other incoherent light sources, the spectral radiance (photon degeneracy number) of our DCL exceeds that of a superluminescent diode (SLD) by one order of magnitude and a light-emitting diode (LED) by six orders of magnitude (see Supplementary Materials). Such high brightness is critical to dynamic imaging with short integration time. Tuning the spatial coherence is more appropriate to suppress coherent artifacts than tuning the temporal coherence, as illustrated in Fig. 6. When illuminated by broadband light with low temporal coherence but high spatial coherence, a meta-hologram creates multiple images whose size scales with the wavelength (see Fig. 6b). An incoherent sum of these images produces a radially extended image with strong edge blurring, as confirmed numerically in Fig. 6e. If the holographic images are offset from the central axis, the image dilation for different wavelengths occurs along a diagonal direction, causing an inhomogeneous edge blurring (see Fig. 6d). In contrast, the edge blurring is less severe and more homogeneous when lowering the spatial coherence of illuminating light for both on-axis and off-axis holography (see Fig. 6a, c). In addition, the temporal coherence tuning is usually done by spectral filtering of a broadband source, which causes a notable change of illumination power.

While the coherent artifacts are strongly suppressed by our method, they cannot be completely removed by lowering the spatial coherence, otherwise, the image blurring would be too severe. Full removal of all coherent artifacts with little loss of spatial resolution is challenging, but may be possible in the future via a combination of minimizing cross-talk and phase dislocation by optimizing the metasurface structure with machine-learning and inverse design, reducing the fabrication defects with high-precision nanofabrication, and removing the residual artifacts with a slight decrease of the spatial coherence of illuminating light.

In summary, our scheme can rapidly and efficiently suppress all coherent artifacts created by different types of meta-holograms. It paves the way for the applications of meta-holograms in dynamic display, augmented reality, optical storage, beam multiplexing, nonlinear holography, and optical manipulation.

## Materials and methods

### Digital meta-hologram design

We design two types of metasurface holograms. In the first one, the phase modulation is obtained via resonant scattering of silicon nanopillars with varying diameter. The second type is based on a geometric Pancharatnam-Berry (PB) phase modulation induced by silicon nanofins of different in-plane orientation (meta-atoms). Using a commercially available finite element method (FEM) solver (COMSOL Multiphysics), we calculate the phase response *φ* of a single nanopillar with diameter *D* and a nanofin with orientation angle *θ*. Periodic boundary conditions are applied, thus neighboring nanopillars (nanofins) are assumed to have an identical diameter *D* (orientation angle *θ*). By varying (*θ*), we obtain the mapping *φ*(*D*) [*φ*(*θ*)]. We use an iterative phase retrieval (IPR) algorithm to find the near-field phase modulation creating a far-field holographic image. The standard method based on the Gerchberg-Saxton (GS) algorithm^47^ generates optical vortices (phase dislocations) in the holographic image. To eliminate such artifacts, the GS algorithm is modified with an initial spherical phase front^50^ and followed by a simulated annealing (SA) routine^39,41^. Finally, we encode the near-field phase profile *φ*_H_, unit cell by unit cell, in a metasurface comprising of 128 × 128 unit cells. Each unit cell contains 2 × 2 nanopillars (nanofins) of the same size (orientation). The nanopillar (nanofin) diameter *D* (orientation angle *θ*) in every unit cell is chosen from the inverse mapping *D*(*φ*) [*θ*(*φ*)]. For the nanopillar hologram, the 2π phase modulation is achieved by varying *D* from 142 nm to 366 nm (see Supplementary Materials). In the nanofin hologram, all nanofins have an identical size but varying orientation. Each fin has a length of 393 nm, a width of 82 nm, and a thickness of 600 nm. The geometric phase *φ* is dictated by the in-plane orientation angle *θ*: *φ* = ±2*θ*, where the ± signs correspond to left- and right-circular polarizations of incident light. With *θ* varying from 0 to π, the phase *φ* modulation covers a 2π range. When illuminated by the linear polarized emission from the DCL, the nanofin hologram generates two images of left and right circular polarizations at different far-field locations. While the design of the meta-hologram phase profile *φ*_H_ is done by tiling unit cells with the pre-calculated phase response of individual meta-atoms, the actual phase response *φ*_A_ of a small meta-hologram with 8 × 8 unit cells is calculated by the FEM with absorbing boundary conditions, and shown in the right panel of Fig. 1b.

### Meta-surface fabrication

The silicon (Si) metasurfaces are fabricated with electron-beam lithography and reactive ion etching. A 600 nm thick amorphous Si film is deposited on a glass substrate using an electron beam evaporator (SKE_A_75). Then, a 100 nm thick electron-beam resist (MicroChem PMMA [polymethyl methacrylate] A2) is spin-coated onto the Si film and patterned with the electron beam writer (Raith E-line, 30 kV). After development in a MIBK & IPA (1:3) solution, 15 nm thick Chromium (Cr) is deposited on the sample using electron beam evaporation (SKE_A_75) and the inverse nano-pattern is transferred to the Cr layer by a lift-off process in a remover PG (Micro Chem). By etching the Si with Sulfur hexafluoride (SF6) and Fluoroform (CHF3) in a flow of 5 sccm and 50 sccm, respectively, the nano-patterns are transferred to the Si membrane. Finally, the Cr mask is removed by immersing the samples in a Cr etchant solution (Aldrich Chemistry) for 30 minutes.

### Spatial coherence tuning of the DCL

The coherence level of the DCL is tuned by translating a lens (L1 in Fig. 2a) inside the cavity along the longitudinal axis. The lens is mounted on a mechanical translation stage with micrometer resolution (Thorlabs MBT616D). When the lens L1 is accurately positioned (Δ*z* = 0 μm) to satisfy the cavity self-imaging condition, lasing occurs in many transverse modes with nearly degenerate loss, and the total emission has a low spatial coherence. Moving the lens L1 from Δ*z* = 0 μm breaks the degeneracy condition and reduces the number of transverse lasing modes. At Δ*z* = 300 μm, nearly all transverse modes stop lasing except one and the spatial coherence of emission is high. By gradually changing Δz from 0 μm to 300 μm, we can continuously vary the number of transverse lasing modes, and accurately tune the degree of spatial coherence from low to high.

### Characterization of spatial coherence

To measure the number of independent transverse lasing modes *N* in the DCL, we direct the lasing emission to a spatial coherence measurement setup. It consists of two lens (L3, L4) with identical focal length *f*, which are arranged in a 4*f* configuration. A ground glass diffuser (Thorlabs DG10-600) is placed in between L3 and L4 at the mutual focal plane (see Supplementary Materials for a schematic and more details). The speckle pattern generated by the diffuser is measured by a CCD camera at the back focal plane of the second lens L4. The intensity contrast *C* of the speckle pattern is defined as *C* = $\sigma _I/\left\langle I \right\rangle$, where $\left\langle I \right\rangle$ is the mean intensity and *σ*_I_ is the standard deviation of intensity fluctuation. *C* is related to the number of independent transverse lasing modes *N* by *C* = $1/\sqrt N$. For different detunings (Δ*z*) of the DCL, the number of transverse lasing modes is estimated from the measured speckle contrast: $N = 1/C^2$.

In order to validate the mutual incoherence of the bright isolated spots in the near-field emission pattern of DCL (Fig. 2b), we measure the spatial pattern of the output beam as it propagates away from the DCL. The diffraction of emission from individual spots causes a spatial overlap of neighboring ones, but no interference fringes are observed in the time-integrated emission pattern, indicating these lasing spots are mutually incoherent.

In the holographic imaging setup, the diameter *L*_I_ of the illuminating beam is larger than the lateral dimension *L*_H_ of a meta-hologram to ensure a uniform intensity illumination. The angular width of the illuminating beam ΔΘ_I_ is inversely proportional to the spatial coherence length *L*_C_: ${\mathrm{{\Delta}}{\Theta}}_{\mathrm{I}} \propto 1/L_{\mathrm{C}}$. The ratio between the illuminating beam area $A_{\mathrm{I}} \propto L_{\mathrm{I}}^2$ and the coherence area $A_{\mathrm{C}} \propto L_{\mathrm{C}}^2$ gives the number of independent spatial modes:

2 $$N = \frac{{A_{\mathrm{I}}}}{{A_{\mathrm{C}}}} = \left( {\frac{{L_{\mathrm{I}}}}{{L_{\mathrm{C}}}}} \right)^2$$

Since the illuminating beam area is larger than the meta-hologram area $A_{\mathrm{H}} = L_{\mathrm{H}}^2$, the effective number of modes *N*_E_ within the meta-hologram is smaller than *N*. The effective number of modes *N*_E_, interacting with the meta-hologram and producing independent holographic images, is given by the ratio of the meta-hologram area and the illumination coherence area:

3 $$N_{\mathrm{E}} = \frac{{A_{\mathrm{H}}}}{{A_{\mathrm{C}}}} = \left( {\frac{{L_{\mathrm{H}}}}{{L_{\mathrm{C}}}}} \right)^2 \propto \left( {\frac{{{\mathrm{{\Delta}}{\Theta}}_{\mathrm{I}}}}{{{\mathrm{{\Delta}}{\Theta}}_{\mathrm{H}}}}} \right)^2$$

where ${\mathrm{{\Delta}}{\Theta}}_{\mathrm{H}} \propto 1/L_{\mathrm{H}}$ is the diffraction angle of the meta-hologram due to its finite size. The effective number of spatial modes in illumination *N*_E_ gives the number of independent holographic images generated at the far-field. In our setup, the ratio $N/N_{\mathrm{E}} = A_{\mathrm{I}}/A_{\mathrm{H}}$ is found to be approximately 8.

## Supplementary information

Supplementary Information

## References

[1] Chen HT, Taylor AJ, Yu NF (2016). A review of metasurfaces: physics and applications. Rep. Prog. Phys..

[2] Glybovski SB (2016). Metasurfaces: from microwaves to visible. Phys. Rep..

[3] Genevet P (2017). Recent advances in planar optics: from plasmonic to dielectric metasurfaces. Optica.

[4] Genevet P, Capasso F (2015). Holographic optical metasurfaces: a review of current progress. Rep. Prog. Phys..

[5] Kamali SM (2018). A review of dielectric optical metasurfaces for wavefront control. Nanophotonics.

[6] Huang LL, Zhang S, Zentgraf T (2018). Metasurface holography: from fundamentals to applications. Nanophotonics.

[7] Jiang Q, Jin GF, Cao LC (2019). When metasurface meets hologram: principle and advances. Adv. Opt. Photonics.

[8] Ni XJ, Kildishev AV, Shalaev VM (2013). Metasurface holograms for visible light. Nat. Commun..

[9] Huang LL (2013). Three-dimensional optical holography using a plasmonic metasurface. Nat. Commun..

[10] Zheng GX (2015). Metasurface holograms reaching 80% efficiency. Nat. Nanotechnol..

[11] Wan WW, Gao J, Yang XD (2017). Metasurface holograms for holographic imaging. Adv. Optical Mater..

[12] Lee GY, Sung J, Lee B (2019). Recent advances in metasurface hologram technologies (Invited paper). ETRI J..

[13] Cui TJ (2014). Coding metamaterials, digital metamaterials and programmable metamaterials. Light. Sci. Appl..

[14] Chen WT (2014). High-efficiency broadband meta-hologram with polarization-controlled dual images. Nano Lett..

[15] Wen DD (2015). Helicity multiplexed broadband metasurface holograms. Nat. Commun..

[16] Segal N (2015). Controlling light with metamaterial-based nonlinear photonic crystals. Nat. Photonics.

[17] Gao LH (2015). Broadband diffusion of terahertz waves by multi-bit coding metasurfaces. Light.: Sci. Appl..

[18] Ye WM (2016). Spin and wavelength multiplexed nonlinear metasurface holography. Nat. Commun..

[19] Qu GY (2020). Reprogrammable meta-hologram for optical encryption. Nat. Commun..

[20] Fang XY, Ren HR, Gu M (2020). Orbital angular momentum holography for high-security encryption. Nat. Photonics.

[21] Yang JJ, Sell D, Fan JA (2018). Freeform metagratings based on complex light scattering dynamics for extreme, high efficiency beam steering. Ann. der Phys..

[22] Liu ZC (2020). Compounding meta‐atoms into metamolecules with hybrid artificial intelligence techniques. Adv. Mater..

[23] Jiang JQ, Fan JA (2019). Global optimization of dielectric metasurfaces using a physics-driven neural network. Nano Lett..

[24] Hemmatyar O (2019). Full color generation with fano-type resonant HfO_2_ nanopillars designed by a deep-learning approach. Nanoscale.

[25] McKechnie, T. S. *Speckle Reduction in Laser Speckle and Related Phenomena* 123–170 (ed Dainty, J. C.) (Springer, 1975).

[26] Cao H (2019). Complex lasers with controllable coherence. Nat. Rev. Phys..

[27] Chriki R (2018). Spatiotemporal supermodes: rapid reduction of spatial coherence in highly multimode lasers. Phys. Rev. A.

[28] Bianco V (2018). Strategies for reducing speckle noise in digital holography. Light. Sci. Appl..

[29] Deng YB, Chu DP (2017). Coherence properties of different light sources and their effect on the image sharpness and speckle of holographic displays. Sci. Rep..

[30] Dubois F, Joannes L, Legros JC (1999). Improved three-dimensional imaging with a digital holography microscope with a source of partial spatial coherence. Appl. Opt..

[31] Lee S (2020). Light source optimization for partially coherent holographic displays with consideration of speckle contrast, resolution, and depth of field. Sci. Rep..

[32] Shin SH, Javidi B (2002). Viewing-angle enhancement of speckle-reduced volume holographic three-dimensional display by use of integral imaging. Appl. Opt..

[33] Dubois F (2004). Partial spatial coherence effects in digital holographic microscopy with a laser source. Appl. Opt..

[34] Golan L, Shoham S (2009). Speckle elimination using shift-averaging in high-rate holographic projection. Opt. Express.

[35] Takaki Y, Yokouchi M (2011). Speckle-free and grayscale hologram reconstruction using time-multiplexing technique. Opt. Express.

[36] Nixon M (2013). Efficient method for controlling the spatial coherence of a laser. Opt. Lett..

[37] Knitter S (2016). Coherence switching of a degenerate VECSEL for multimodality imaging. Optica.

[38] Kim K (2019). Electrically pumped semiconductor laser with low spatial coherence and directional emission. Appl. Phys. Lett..

[39] Seldowitz MA, Allebach JP, Sweeney DW (1987). Synthesis of digital holograms by direct binary search. Appl. Opt..

[40] Wyrowski F, Bryngdahl O (1989). Speckle-free reconstruction in digital holography. J. Optical Soc. Am. A.

[41] Feldman MR, Guest CC (1989). Iterative encoding of high-efficiency holograms for generation of spot arrays. Opt. Lett..

[42] Senthilkumaran P, Wyrowski F, Schimmel H (2005). Vortex stagnation problem in iterative fourier transform algorithms. Opt. Lasers Eng..

[43] Maycock J (2007). Reduction of speckle in digital holography by discrete fourier filtering. J. Optical Soc. Am. A.

[44] Lee GY (2018). Complete amplitude and phase control of light using broadband holographic metasurfaces. Nanoscale.

[45] Overvig AC (2019). Dielectric metasurfaces for complete and independent control of the optical amplitude and phase. Light. Sci. Appl..

[46] Arnaud JA (1969). Degenerate optical cavities. Appl. Opt..

[47] Gerchberg RW, Saxton WO (1972). A practical algorithm for the determination of phase from image and diffraction plane pictures. Optik.

[48] Bomzon Z (2002). Space-variant Pancharatnam–berry phase optical elements with computer-generated subwavelength gratings. Opt. Lett..

[49] Cohen E (2019). Geometric phase from Aharonov–Bohm to Pancharatnam–berry and beyond. Nat. Rev. Phys..

[50] Pang H (2019). Speckle-reduced holographic beam shaping with modified Gerchberg–Saxton algorithm. Opt. Commun..

