# Supplementary material for: Suppressing meta-holographic artifacts by laser coherence tuning
Source: Light Sci Appl. 2021 May 19;10:104. doi: 10.1038/s41377-021-00547-0 (PMC8134448; doi:10.1038/s41377-021-00547-0)
Supplement: Supplementary file 1 — Supplementary Information [file 41377_2021_547_MOESM1_ESM.pdf]

Supplementary information for "Suppressing meta-holographic artifacts by laser coherence tuning"

Yaniv Eliezer[1,†], Geyang Qu[2,†], Wenhong Yang[2], Yujie Wang[2], Hasan Yilmaz[1], Shumin Xiao[2,*], Qinghai Song[2,*], and Hui Cao[1,*]

[1]Department of Applied Physics, Yale University, New Haven, Connecticut 06520, USA
[2]Ministry of Industry and Information Technology Key Lab of Micro-Nano Optoelectronic Information System, Shenzhen Graduate School, Harbin Institute of Technology, Shenzhen, 518055, China
[*]E-mail: shumin.xiao@hit.edu.cn, qinghai.song@hit.edu.cn, hui.cao@yale.edu
[†]These authors contributed equally to this work.

ABSTRACT

This document provides supplementary information to “Suppressing meta-holographic artifacts by laser coherence tuning”. We discuss the meta-hologram design and modeling in section (I). Section (II) explains the electromagnetic cross-talk in meta-holograms, and section (III) provides a sensitivity analysis of the meta-surface hologram. Section (IV) presents a detailed view of the optical setup. Section (V) describes the lasing emission power and section (VI) covers the emission spectrum and temporal coherence of the degenerate cavity laser (DCL). In section (VII) we provide additional results of coherent artifacts suppression. Finally, we present the theoretical models of the holographic image sharpness and contrast-to-noise ratio (CNR) in sections (VIII) and (IX).

(I) Meta-hologram design and modeling

Computer generated holography (CGH) and phase encoding

The first step in designing a digital hologram is computing the near-field phase profile that produces the desired holographic image in the far-field. The digitized intensity matrix of the holographic image is fed into a standard iterative phase retrieval (IPR) algorithm[1]. If the initial phase pattern is random, optical vortices (phase dislocations) are introduced to the holographic image. To exclude the optical vortices, the initial phase pattern is set to a spherical phase front, and simulated annealing is incorporated in the iterative routine[2,3]. Finally, the nonlinear search converges to a discrete phase profile $\phi_{i,j}$.

Resonant phase modulation meta-hologram design

The first type of meta-holograms is based on resonant scattering of silicon nanopillars (meta-atoms).

The electromagnetic phase response ϕ of a single nanopillar is numerically calculated using a commercial finite-element-method (FEM) solver (COMSOL Multiphysics) for a broad range of pillar diameters D. Periodic boundary conditions are used, under the assumption that neighboring nanopillars have an identical diameter. The resulting function $\phi(D)$ maps the nanopillar diameter D to the phase modulation ϕ. Figure S1 shows the calculated $D(\phi)$ of a single silicon nano-pillar at the optical wavelength of 1055 nm.

Every element of the designed phase hologram $\phi_{i,j}$ corresponds to a unit cell comprised of 2×2 nanopillars with the same diameter. The inverse mapping function $D(\phi)$ is used to set the nanopillar diameter D in each unit cell $D(\phi_{i,j})$. As D varies from one unit cell to the next, nanopillars in adjacent unit cells might have different diameters. Their near-field interactions would differ from the case of identical nanopillars, which is assumed in the calculation of $\phi(D)$ for a single nanopillar with periodic boundary conditions. Such difference makes the actual phase response ϕ_A deviate from the designed one ϕ_H, creating artifacts in the holographic image.

The selection of 2×2 nanopillars in a unit cell reduces the cross-talk coherent artifacts. To show this improvement, we calculated the actual phase modulation of a meta-hologram with 8x8 unit cells. As shown in Fig. S2 below, each unit cell contains a single nanopillar in **a**, or four nanopillars in **b**. While both phase distributions **c,d** deviate from the designed one in **e**, the deviation for the single-pillar unit cell in **c** is notably larger than the four-pillars unit cell in **d**.

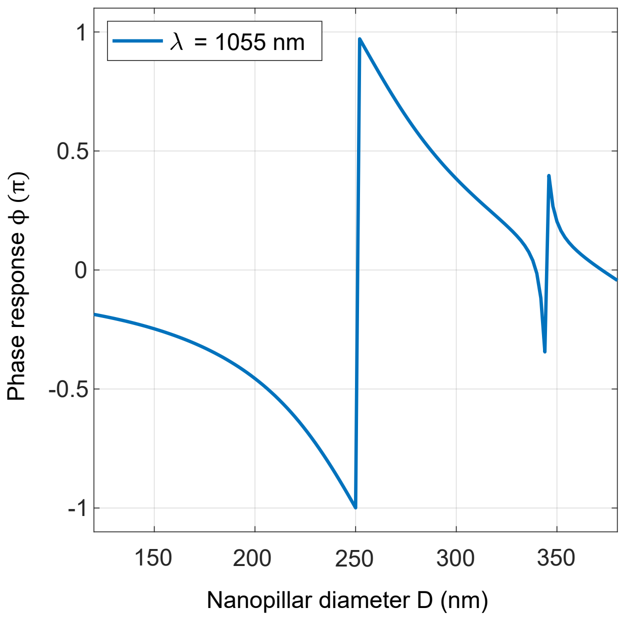

Figure S1. Simulated holographic images. Calculated phase response ϕ of a single silicon nanopillar as a function of its diameter D at wavelength 1055 nm.

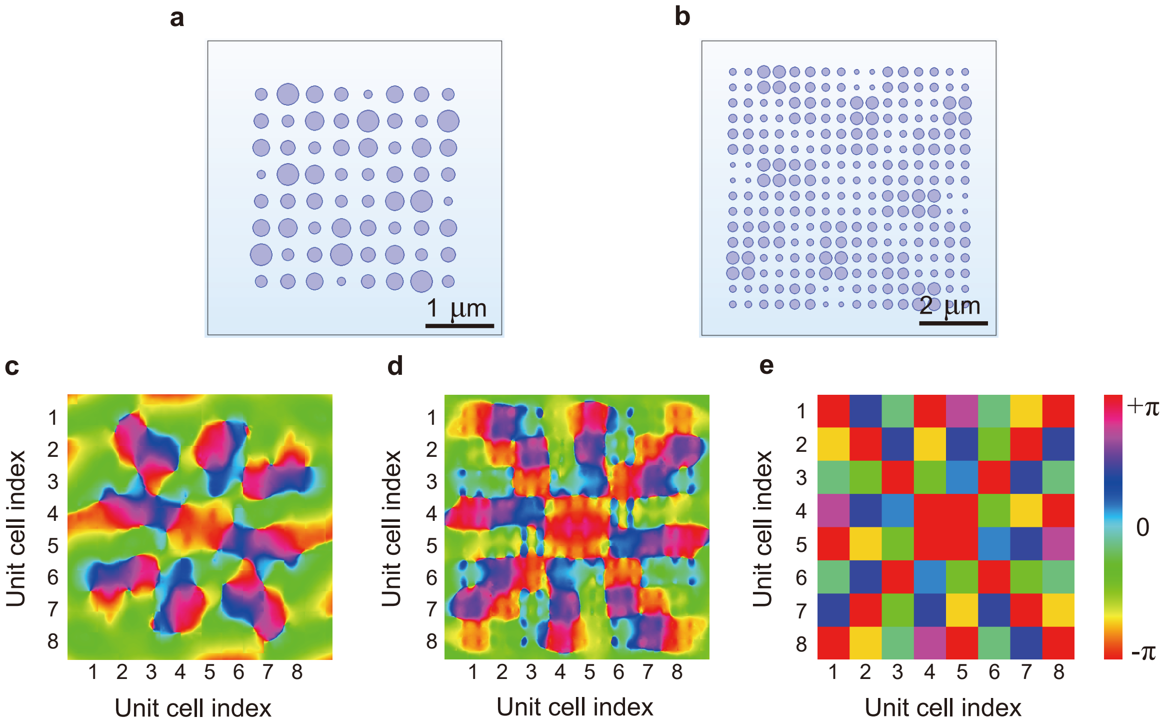

Figure S2. Cross-talk analysis for different unit cell size. a,b Layout of a meta-surface containing 8×8 unit cells, each cell containing 1 nanopillar (**a**) or 2×2 nanopillars (**b**). **c,d** Numerically calculated phase modulation for **a,b**, showing stronger deviation from the design **e** with 1-nanopillar unit cell (**c**) than 4-nanopillars unit cell (**d**).

Geometric Pancharatnam-Berry phase meta-hologram design

In a metasurface, local birefringence can be induced by anisotropic scatterers (meta-atoms). We use silicon nanofins of length 393 nm, width 82 nm and thickness 600 nm. The geometric phase ϕ is determined by the in-plane orientation angle θ of the nanofin and circular polarization state of the illuminating light. The phase delay ϕ for incident left-circular polarization (LCP) and diffracted right-circular polarization (RCP) is 2θ, and that for incident RCP and diffracted LCP is -2θ. As θ varies from 0 to π, the phase ϕ modulation covers a 2π range. Using the FEM (COMSOL Multiphysics), we calculate $\phi(\theta)$ for a single silicon nanofin with periodic boundary conditions. Figure S3a shows the phase delay $\phi(\theta)$ for two circular polarizations.

Every element of the designed phase hologram $\phi_{i,j}$ corresponds to a unit cell comprised of 2×2 nanofins with the same orientation angle θ. The inverse mapping function $\theta(\phi)$ is used to set the in-plane orientation angle θ of nanofins in each unit cell $\theta(\phi_{i,j})$. As θ varies from one unit cell to the next, nanopillars in adjacent unit cells might have different orientations. Their near-field interactions would differ from nanofins oriented with the same angle, which is assumed in the calculation of $\phi(\theta)$ for a single nanofin with periodic boundary conditions. Such difference makes the actual phase response ϕ_A deviate from the designed one ϕ_H, generating artifacts in the holographic image.

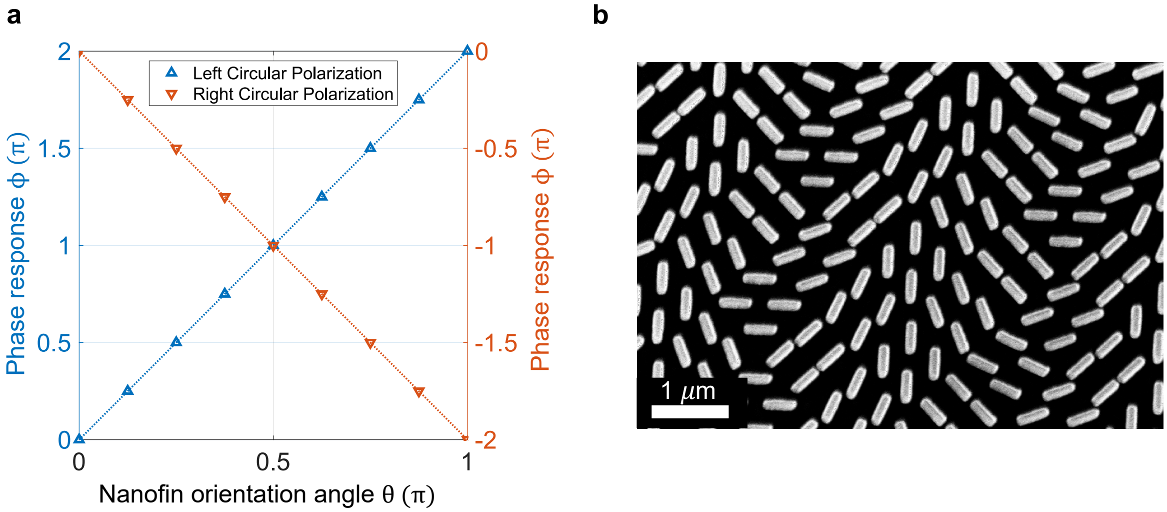

Figure S3. Geometric Pancharatnam-Berry phase meta-hologram. **a** Calculated phase delay ϕ of a single silicon nanofin as a function of its in-plane orientation angle θ for left circular and right circular polarizations at wavelength 1055 nm. The Periodic boundary conditions are applied. **b** Scanning electron microscope image of part of a metasurface comprised of 128×128 unit cells. Each unit cell contains 2×2 silicon nanofins with varying orientation angles, that acquire different geometric phase delays. All nanofins, lying on a glass substrate, have an identical length of 393 nm, width of 82 nm and thickness of 600 nm.

In our holographic imaging experiment, the meta-hologram is illuminated by the linear polarized emission from the DCL. The illuminating light is a linear superposition of LCP and RCP, which acquire different geometric phase delays from the nanofin hologram. Consequently two holographic images of different circular polarizations are created in the far field at different locations.

(II) Electromagnetic cross-talk in meta-holograms

The meta-atoms are closely packed in a meta-hologram, the spacing of neighboring meta-atoms is comparable to their size. Hence, their near-field interactions are significant, and their electromagnetic phase response is collective. Such collective response depends on the size (orientation) of the adjacent nanopillars (nanofins), which are assumed to be identical in the simulation of a single meta-atom with periodic boundary conditions. In the meta-hologram, the nanopillar diameter D (nanofin orientation angle θ) varies from one unit cell to the next, thus the nanopillars (nanofins) in neighboring cells may have different D (θ). Their near-field interactions differ from those between identical meta-atoms, causing the collective phase response to deviate from the calculated one used in the hologram design[4,5]. This deviation causes a distortion of the holographic image, introducing strong intensity variations.

To illustrate such artifacts, we design the holograms free of optical vortices (phase dislocations) by applying a modified Gerchberg-Saxton (GS) routine that eliminates most of the optical vortices[2,3,6]. Fig. S4a shows the simulated image of a hologram with phase encoded by the standard GS algorithm. There are many optical vortices across the image, causing strong intensity fluctuations. In Fig. S4b, the far-field image of a hologram encoded by the modified GS algorithm appears free of optical vortices, with a relatively uniform intensity distribution. However, such uniformity disappears when near-field interactions of neighboring meta-atoms are taken into account in a full-wave simulation, as shown in Fig. S4c. The severe artifacts are caused by the cross-talk, as the fabrication defects are ignored in the simulation.

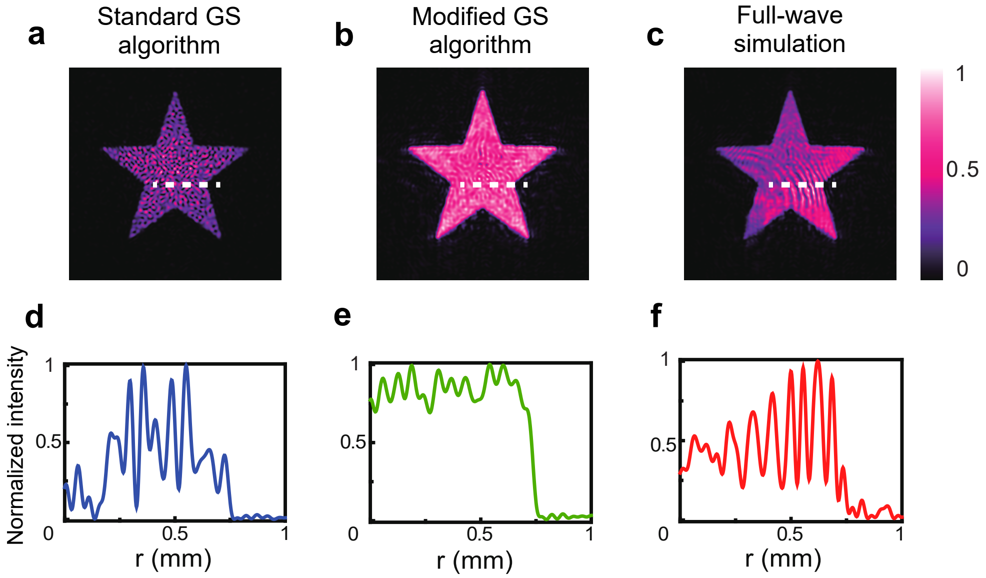

Figure S4. Coherent artifacts from optical vortices and meta-atom coupling. **a,b** Simulated far-field holographic images generated using the standard and modified GS algorithms respectively. **c** Far-field holographic image encoded by the modified GS algorithm and simulated with a full-wave calculation taking into account the near-field coupling in a meta-hologram with 128×128 unit cells. The calculation takes an immense computational effort. **d,e,f** 1D intensity profiles obtained from the top holographic images **a,b,c** along the white dashed line respectively. The intensity fluctuation in **c** is much larger than that in **b**, due to strong coupling between meta-atoms (cross-talk).

Next we show experimentally the magnitude of cross-talk effects for our meta-holograms. As an example, Fig. S5 shows an measured image of a star generated by a nano-pillar hologram labeled A in the left panel. The middle panel is a closeup of the intensity fluctuation inside the star image. To check such artifacts result from near-field coupling of nanopillars or fabrication defects, we fabricate a second meta-hologram of identical structure. The near-field coupling between nanopillars is unchanged, leading to same artifacts. However, fabrication defects vary from sample to sample, and the resulting artifacts would be different. The right panel is a closeup of the image generated by the second meta-hologram labeled B, which displays almost the same intensity fluctuation as the first one (middle panel). Such resemblance confirms that the artifacts mainly originate from the deterministic near-field interactions of meta-atoms.

Even with phase dislocations removed, the contrast of intensity fluctuations in Fig. S5 is about 0.3. This value is much higher than the contrast of speckle noise in classical holographic images. To have a speckle contrast of 0.3, the random phase fluctuation in a classical hologram would have a magnitude of π, which is much larger than the real value. Hence, the meta-holograms have much more severe coherent artifacts as a result of strong near-field coupling between closely packed meta-atoms.

To remove such artifact, the near-field interactions between meta-atoms must be accurately accounted for in the hologram design. This can be done, in principle, by calculating the collective phase response of an entire metasurface. In reality, a numerical simulation of a meta-hologram containing tens of thousands of meta-atoms is computationally demanding even for a single realization, and any iterative optimization scheme requires repetitive calculation of the entire meta-hologram, rendering this approach unfeasible. Alternatively, one may increase the spatial separation of meta-atoms to reduce their near-field interactions, or construct large unit cells with many meta-atoms of same size/orientation so that most meta-atoms have neighbors identical to themselves. These approaches, however, will significantly increase the meta-hologram footprint and reduce the viewing angle[5].

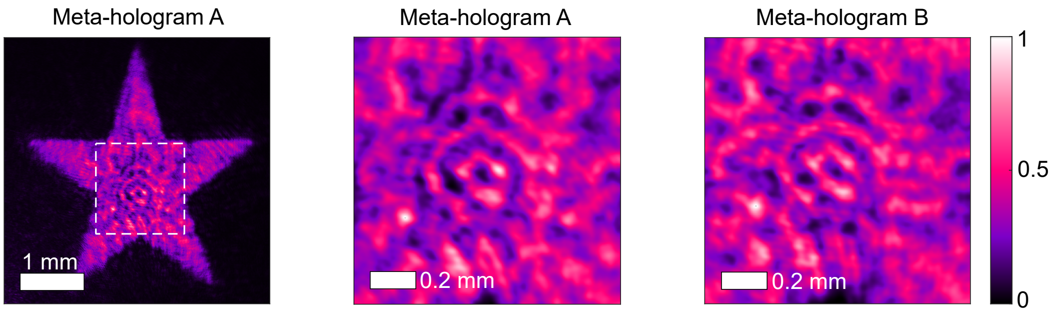

Figure S5. Coherent artifacts of meta-holograms. **Left:** Experimentally measured image of a star created by a nanopillar hologram. The illuminating beam is from a continuous-wave laser of wavelength λ = 1064 nm. Although the image is designed to be a smooth star and optical vortices are already eliminated from the computer-generated hologram, strong intensity modulation is seen across the star. **Center:** A closeup of the intensity pattern within the dashed white square in the left image, revealing the detail of intensity fluctuation. **Right:** A closeup of the far-field intensity pattern for a second meta-hologram **B** with the same design as the first one **A**. They produce nearly identical intensity variations, indicating the artifact is caused mainly by the deterministic interaction (cross-talk) of meta-atoms instead of random fabrication defects.

(III) Meta-hologram sensitivity analysis

Here we investigate how sensitive our meta-holograms to variations in illumination wavelength, angle of incidence, and number of unit cells. The nanopillar hologram is studied as an example. The nanopillars have 8 different diameters, corresponding to 8 phase levels. At the design wavelength λ = 1055 nm, the 8 phase values are uniformly distributed over a 2π range for normal incidence of light. We calculate the change of phase delay with wavelength detuning. At normal incidence, the phase delay increases with λ, as shown in Fig. S6a. The increase is almost linear with a slope of the order 0.01 π nm^{-1}. A slight difference in the slope for different pillar diameter leads to a non-uniform distribution of 8 phase levels, and the total range of phase modulation deviates from 2π. For example at λ = 965 nm, the 8 nanopillars only covers a range of 1.6π.

Next we fix the illumination wavelength λ = 1055 nm, and vary the angle of incidence α. Figure S6b shows the calculated phase response of nanopillars with 8 different diameters as a function of α. The phase delay varies slightly as the incident angle changes from -10 ° to 10 °. The overall magnitude of phase change for individual nanopillars is less than 0.4π. Note that the α range (-10 °, 10 °) is larger than the angular spread of emission of our degenerate cavity laser.

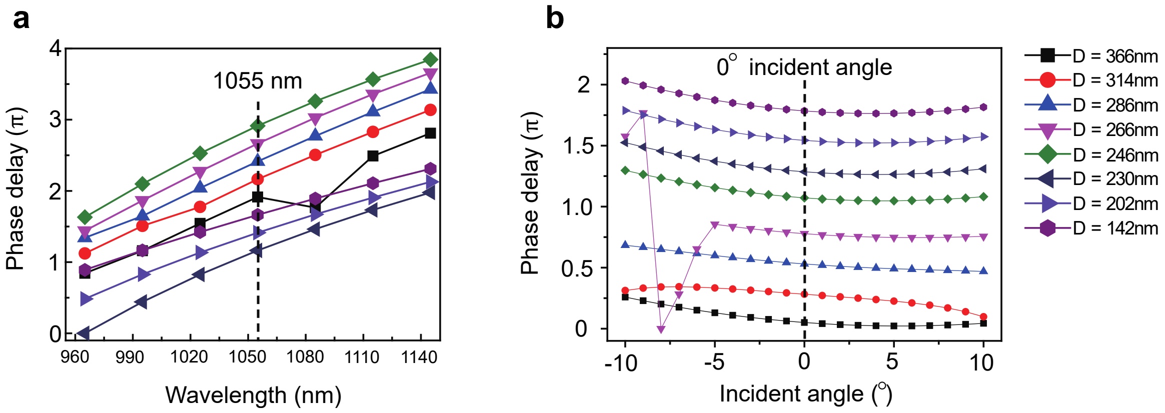

Figure S6. Sensitivity of nanopillar phase response to illumination wavelength and incident angle. **a** Calculated phase delays of 8 nanopillars with diameters D = 142 nm to 366 nm as a function of illumination wavelength λ. The increase of phase delay with wavelength is approximately linear, and the slope varies with the pillar diameter. **b** Calculated phase delay vs. the incident illumination angle α for nanopillars with 8 different diameters D.

Finally, we examine how the meta-hologram size, i.e., the number of unit cells, influence the image quality. In general, a larger meta-hologram size leads to a higher resolution. We simulate the holographic image with different numbers of unit cells. The meta-hologram is designed with the standard GS algorithm. As shown in Fig. S7, finer details in the holographic image of USAF resolution test chart are resolved with an increasing number of unit cells.

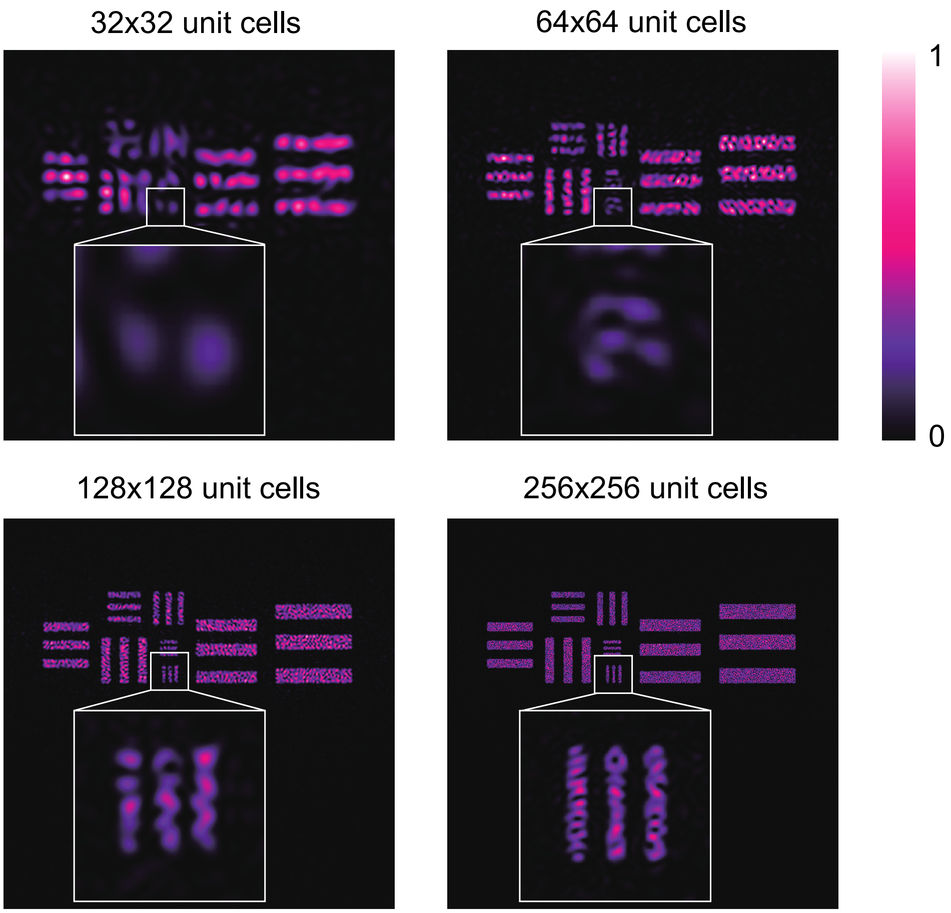

Figure S7. Sensitivity of image quality to meta-hologram size. USAF resolution test chart holographic images generated by meta-holograms with different number of unit cells. With 32×32 unit cells, the test chart details are poorly resolved. As the number of unit cells increases, the image resolution improves.

(IV) Optical setup

Our experimental setup, sketched in Fig. S8, consists of three arms. The central arm contains the degenerate cavity laser (DCL). The DCL is comprised of a broad-area (450 μm diameter) electrically-pumped vertical external cavity surface emitting laser (VECSEL) module (VL), two plano-convex lenses (L1, L2) with focal length 13.2 mm and 103 mm, and an output coupler (OC) with 95 % reflectivity. L1 is mounted on a mechanical translation stage with micrometer-scale resolution (Thorlabs MBT616D).

To measure the number N of independent transverse lasing modes in the cavity, we use two mirrors (SM, M1) to direct the emission into a second arm for spatial coherence characterization. This arm consists of two lens (L3, L4) with an identical focal length (150 mm) arranged in a $4f$ configuration. A ground glass diffuser (D1) from Thorlabs (DG10-600) is placed at the mutual focal plane in between L3 and L4. The speckle pattern, produced by the diffuser, is measured at the back focal plane of L4 by a CCD camera (CAM2). The speckle intensity contrast is evaluated as $C \equiv \sigma_I / \langle I \rangle$, where $\langle I \rangle$ is the spatially averaged intensity, and σ_I is the standard deviation of intensity fluctuation around the mean. The number of spatial modes N is estimated from the speckle contrast by $N = 1/C^2$.

The third holographic imaging arm contains a pair of Mitutoyo NIR objective lens (O1, O2). The back focal plane of O1 coincides with the front focal plane of O2, at which the meta-hologram (MH) is positioned. The far-field emission from the DCL illuminates the meta-hologram. The holographic image is collected by O2 and projected by a tube lens (L6) onto a CCD camera (CAM1).

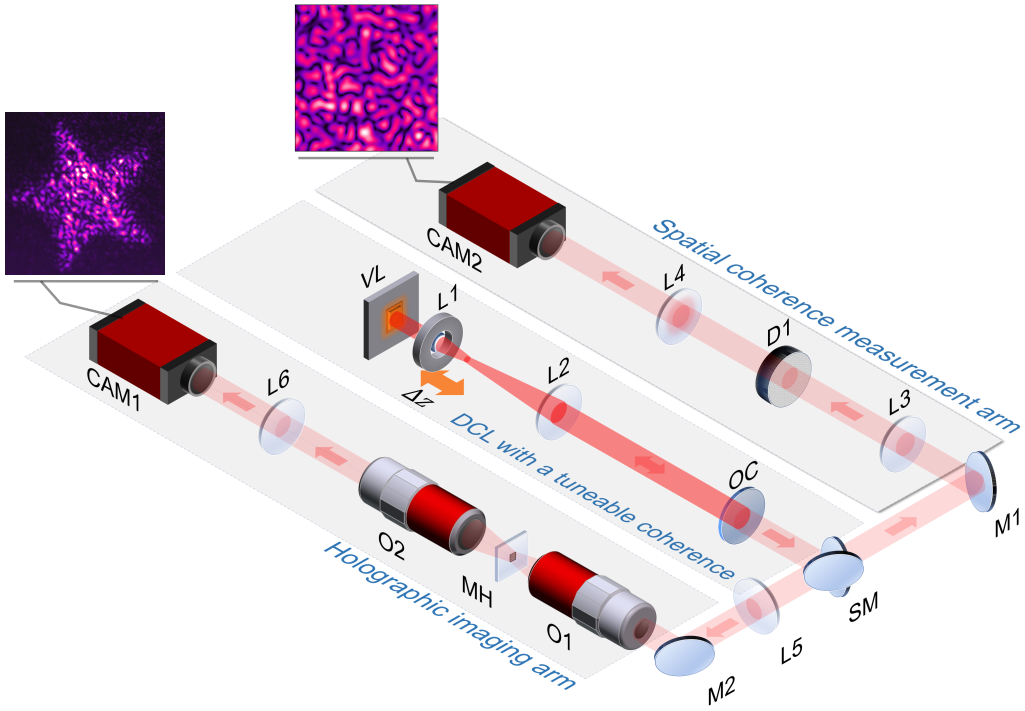

Figure S8. Optical setup. See the text for a detailed description. (DCL) degenerate cavity laser, (VL) vertical external cavity surface emitting laser device, (L) lens, (Δz) axial translation of L1, (OC) output coupler, (SM) switchable Mirror, (M) mirror, (D) ground glass diffuser, (O) objective lens, (MH) meta-hologram, (BS) beam-splitter, (CAM) CCD camera. The insets show a meta-holographic image and a speckle pattern recorded by two cameras.

(V) Laser emission power

Figure S9 shows the measured DCL output power as a function of the number of transverse lasing modes. As we break the loss degeneracy of the transverse lasing modes by moving the intracavity lens (L1), the number of transverse modes N decreases, and the degree of spatial coherence increases. When N decreases from 300 to 10, the total emission power changes from 108 mW to 64 mW. The 30 times reduction in N only results in 40 % decrease of power. An alternative way of increasing the spatial coherence by spatial filtering of the emission outside the DCL, would cause a severe power loss, as the filtered power scales linearly with the number of spatial modes N (see the red dashed line in Figure S9).

To quantitatively compare the brightness of our DCL to other illumination sources such as light-emitting diodes (LEDs) and super-luminescent diodes (SLDs), we calculate the photon degeneracy δ by estimating the number of photons per coherence volume[7]. When the number of transverse lasing modes N in our DCL is close to 1, the photon degeneracy δ is in the order of 10^5. As N increases to 300, δ drops by one order of magnitude to 10^4. Note that the change in the temporal coherence is negligible [see (VI) for more details]. For comparison, LEDs have δ in the order of 10^{-2}, and SLDs[8] in the order of 10^3. Hence, the photon degeneracy of our DCL is at least one order of magnitude higher than the SLDs.

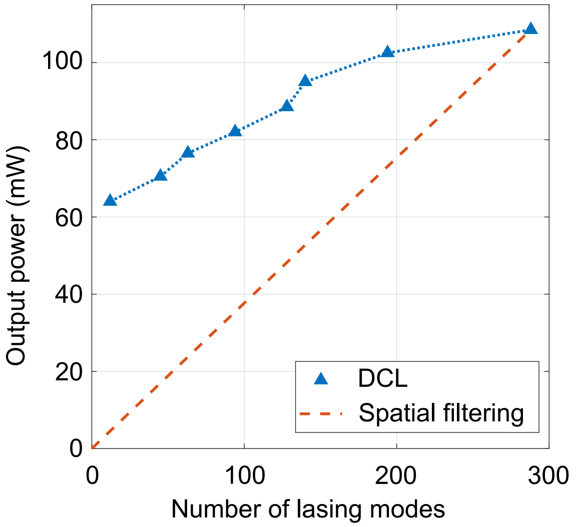

Figure S9. DCL emission power vs. the number of transverse lasing modes. The measured DCL output power increases sublinearly with the number of transverse lasing modes N (blue triangles). Spatial filtering of the DCL emission outside the cavity could also tune the spatial coherence, but at the expense of severe power loss, as the filtered power scales linearly with the number of filtered modes N (red dashed line).

(VI) DCL emission spectrum and temporal coherence

One unique feature of our DCL is that the temporal coherence of emission is nearly constant, while the spatial coherence is tuned by cavity misalignment. The temporal coherence length is inversely proportional to the spectral width of the DCL emission. We measure the emission spectrum of the DCL with different numbers of transverse lasing modes N, using a spectrometer (ACTON SP500) with a wavelength resolution of $\sim$ 0.1 nm. As shown in Fig. S10, the measured spectrum of the DCL far-field emission with $N \simeq 10$ is nearly identical to that with $N \simeq 800$. The spectral full-width-at-half-maximum (FWHM) $\Delta\lambda$ is 3.1 ± 0.1 nm for $N_{\mathrm{E}} \simeq 10$ and 3.0 ± 0.1 nm for $N_{\mathrm{E}} \simeq 800$. Hence, the temporal coherence length remains constant as we tune the DCL, confirming that our method of removing coherent artifacts relies primarily on varying the spatial coherence of the DCL.

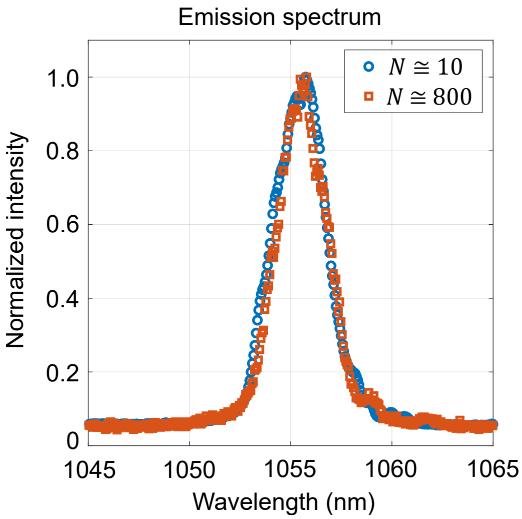

Figure S10. DCL emission spectrum. Blue circles represent the experimentally measured DCL far-field emission spectrum with $N \cong 10$ transverse lasing modes (high spatial coherence). Red squares denote the measured DCL spectrum with $N \cong 800$ transverse lasing modes (low spatial coherence). The full-width-at-half-maximum (FWHM) $\Delta\lambda$ of both spectra is approximately the same, indicating that the temporal coherence length (inversely proportional to $\Delta\lambda$) remains constant during the tuning of spatial coherence.

(VII) Coherent artifacts suppression

In the main text, Fig. 3a shows how artifacts from both meta-atom interactions and optical vortices in a nanopillar hologram are removed by lowering the spatial coherence of illuminating light from the DCL. Here Fig. S11 provides an additional demonstration of coherent artifact suppression for a nanopillar hologram without optical vortices. Under a coherent illumination (the effective number of spatial modes illuminating the hologram $N_E \cong 1$), the holographic image in the left panel of Fig. S11 clearly displays intensity fluctuations across the star, despite the optical vortices are already eliminated in the hologram design process. Increasing the number of spatial modes that illuminate the hologram to $N_E = 15$ greatly reduces the intensity modulation, with a slight degradation of the edge sharpness, as seen in the right panel of Fig. S11.

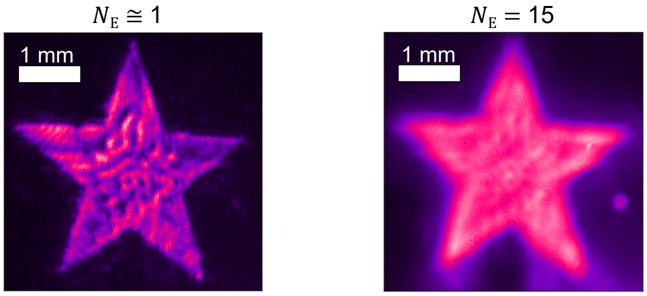

Figure S11. Removing coherent artifact by lowering the spatial coherence of illumination. The meta-hologram contains 128×128 unit cells, each having 2×2 Si nano-pillars of same diameter. The phase dislocations are already removed in the hologram design process. It is illuminated by the DCL emission. By misaligning the DCL, the effective number of spatial modes illuminating the meta-hologram N_E varies. **Left:** when the illuminating light has high spatial coherence ($N_E \simeq 1$), the holographic image of a star displays strong intensity modulation due to near-field interaction of nanopillars. **Right:** Once the degree of spatial coherence is optimized for illumination ($N_E = 15$), the intensity distribution inside the star becomes smooth, while its edge is slightly blurred.

(VIII) Theoretical model of holographic image sharpness

We theoretically investigate the dependence of edge sharpness S on the effective number of spatial modes N_{E} that illuminate the meta-hologram. For $N_{\mathrm{E}} = 1$, the point spread function (PSF) of our holographic imaging setup is approximated by a Gaussian function $I_{\mathrm{P}}(r)$. Its width w_{P} is inversely proportional to the lateral dimension of the meta-hologram. For $N_{\mathrm{E}} > 1$, an incoherent sum of laterally offset images broadens the edge intensity profile. Such broadening is described by a Gaussian function $I_{\mathrm{C}}(r)$ of width w_{C} proportional to the angular spread of incident light. In the case of the DCL illumination, w_{C} scales as $\sqrt{N_{\mathrm{E}}-1}$. The effective PSF $I_{\mathrm{E}}(r)$ can be approximated by the convolution of $I_{\mathrm{P}}(r)$ and $I_{\mathrm{C}}(r)$: $I_{\mathrm{E}}(r) = I_{\mathrm{P}}(r) * I_{\mathrm{C}}(r)$. $I_{\mathrm{E}}(r)$ is a Gaussian function of width $w_{\mathrm{E}} = \sqrt{{w_{\mathrm{P}}}^2 + {w_{\mathrm{C}}}^2}$.

The edge sharpness is defined as $S = (r_{10\%} - r_{90\%})^{-1}$, where $r_{10\%}$ and $r_{90\%}$ are the spatial coordinates of 10 % and 90 % of the maximum intensity in the 1D intensity profile across an edge of the holographic image. With the Gaussian PSF $I_{\mathrm{E}}(r)$, S can be expressed as:

$$S(w_{\mathrm{C}}) = \frac{1}{\sqrt{2}\sqrt{{w_{\mathrm{P}}}^2 + {w_{\mathrm{C}}}^2}\left[\sqrt{\ln\left(\frac{1}{0.1}\right)} - \sqrt{\ln\left(\frac{1}{0.9}\right)}\right]}. \tag{1}$$

Substituting the dependence of w_{C} on N_{E}, we get:

$$S(N_{\mathrm{E}}) = \frac{C_0}{\sqrt{{w_{\mathrm{P}}}^2 + C_1(N_{\mathrm{E}}-1)}}, \tag{2}$$

where C_0 is a prefactor, and C_1 is a scaling constant relating the lateral shift of far-field holographic image to the tilt of the illuminating light incident angle.

(IX) Theoretical model of contrast-to-noise ratio

The contrast-to-noise ratio (CNR) for a holographic image of the USAF test chart is:

$$CNR = \frac{\langle I_{\mathrm{S}}\rangle - \langle I_{\mathrm{B}}\rangle}{\sigma_{\mathrm{S}}} = \frac{1 - \langle I_{\mathrm{B}}\rangle/\langle I_{\mathrm{S}}\rangle}{\sigma_{\mathrm{S}}/\langle I_{\mathrm{S}}\rangle}, \tag{3}$$

where $\langle I_{\mathrm{S}}\rangle$ ($\langle I_{\mathrm{B}}\rangle$) is the spatially averaged intensity of the bright bars (dark background gaps), and σ_{S} is the standard deviation of the intensity fluctuation within the bars. The denominator $\sigma_{\mathrm{S}}/\langle I_{\mathrm{S}}\rangle$ is the intensity contrast which scales as $\sqrt{N_{\mathrm{E}}}$, as shown in Fig. 4a of the main text. Similar to the analysis in (VIII), the background intensity $I_{\mathrm{B}}(r)$ is modeled as the tail of Gaussian PSF $I_{\mathrm{E}}(r)$ in the gap between adjacent bars with edges at $r = \pm d/2$, where d represents the gap width. The mean intensity $\langle I_{\mathrm{B}}\rangle$ can be obtained by integrating $I_{\mathrm{B}}(r)$ over the gap $[-d/2, +d/2]$:

$$\langle I_{\mathrm{B}}\rangle = \frac{1}{d}\int_{-d/2}^{+d/2} I_{\mathrm{B}}(r)dr = \sqrt{\frac{\pi}{2}}\frac{N_{\mathrm{E}}}{d}\left[{w_{\mathrm{P}}}^2 + C_1(N_{\mathrm{E}}-1)\right]^{1/2}\mathrm{erf}\left(\frac{d}{\sqrt{2}\sqrt{{w_{\mathrm{P}}}^2 + C_1(N_{\mathrm{E}}-1)}}\right). \tag{4}$$

Since $\langle I_{\mathrm{S}}\rangle$ scales linearly with the effective number of spatial modes N_{E}, the prefactor N_{E} cancels out in the ratio $\langle I_{\mathrm{B}}\rangle/\langle I_{\mathrm{S}}\rangle$. We further simplify Eq. (4) by assuming w_{P} is negligible compared to w_{C}, leading to the following expression of the CNR:

$$CNR(N_{\mathrm{E}}) = \frac{1 - \langle I_{\mathrm{B}}\rangle/\langle I_{\mathrm{S}}\rangle}{\sigma_{\mathrm{S}}/\langle I_{\mathrm{S}}\rangle} \approx \frac{1 - A_0\sqrt{N_{\mathrm{E}}}\mathrm{erf}\left(\frac{A_1}{\sqrt{N_{\mathrm{E}}}}\right)}{A_2/\sqrt{N_{\mathrm{E}}}} = B_0\left(\sqrt{N_{\mathrm{E}}} - B_1 N_{\mathrm{E}}\mathrm{erf}\left[\frac{B_2}{\sqrt{N_{\mathrm{E}}}}\right]\right), \tag{5}$$

where B_0, B_1, B_2 are constants related to A_0, A_1, A_2. Their values are obtained by fitting the experimentally measured CNR.

For each spatial frequency $k = 1/d$, we solve Eq. (5) numerically to find the optimal $N_{\mathrm{E}}^{(max)}$ at which the CNR reaches the maximum. The maximum of CNR at an intermediate N_{E} can be understood as follows. At small N_{E}, the first term in Eq. (5) dominates, and its growth with N_{E} leads to the rise of CNR. As N_{E} further increases, the second term in Eq. (5) becomes significant and eventually causes the drop of CNR. The optimal $N_{\mathrm{E}}^{(max)}$ at the CNR maximum depends on the spatial frequency k. At higher k, the feature size d is smaller, and $\langle I_{\mathrm{B}}\rangle$ is stronger, therefore the CNR maximum appears at a lower $N_{\mathrm{E}}^{(max)}$.

References

1. Gerchberg, R. W. A practical algorithm for the determination of phase from image and diffraction plane pictures. *Optik* **35**, 237–246 (1972).
2. Seldowitz, M. A., Allebach, J. P. & Sweeney, D. W. Synthesis of digital holograms by direct binary search. *Appl. Opt.* **26**, 2788–2798, 10.1364/AO.26.002788 (1987).
3. Feldman, M. & Guest, C. Iterative encoding of high-efficiency holograms for generation of spot arrays. *Opt. letters* **14 10**, 479–81 (1989).
4. Yang, J., Sell, D. & Fan, J. A. Freeform metagratings based on complex light scattering dynamics for extreme, high efficiency beam steering. *Annalen der Physik* **530**, 1700302 (2018).
5. Huang, L., Zhang, S. & Zentgraf, T. Metasurface holography: from fundamentals to applications. *Nanophotonics* **7**, 1169–1190 (2018).
6. Pang, H., Liu, W., Cao, A. & Deng, Q. Speckle-reduced holographic beam shaping with modified gerchberg–saxton algorithm. *Opt. Commun.* **433**, 44–51 (2019).
7. Mandel, L. & Wolf, E. *Optical coherence and quantum optics* (Cambridge university press, 1995).
8. Hitzenberger, C., Danner, M., Drexler, W. & Fercher, A. Measurement of the spatial coherence of superluminescent diodes. *J. Mod. Opt.* **46**, 1763–1774 (1999).
